# Guidelines for quality control of PET/CT scans in a multicenter clinical study

**DOI:** 10.1186/s40658-017-0190-7

**Published:** 2017-09-18

**Authors:** Ivalina Hristova, Ronald Boellaard, Paul Galette, Lalitha K. Shankar, Yan Liu, Sigrid Stroobants, Otto S. Hoekstra, Wim J.G. Oyen

**Affiliations:** 1 Department of Nuclear Medicine, Radboud University Medical Centre, Geert Grooteplein-Zuid 10, 6525 GA Nijmegen, The Netherlands; 2 University Medical Center Groningen, University of Groningen, Groningen, The Netherlands; 3 The Institute of Cancer Research, London, UK; 4 European Organization for Research and Treatment of Cancer, Imaging Group, Brussels, Belgium; 5 GSK, Experimental Medicine Imaging, Upper Providence, PA USA; 6 Division of Cancer Treatment and Diagnosis National Cancer Institute, Bethesda, MD USA; 7 European Organization for Research and Treatment of Cancer, Headquarters, Brussels, Belgium; 8 Molecular Imaging Center Antwerp (MICA), Faculty of Medicine and Health Sciences, University of Antwerp, Wilrijk, Belgium; 9 Department of Radiology & Nuclear Medicine, VU University Medical Centre, Amsterdam, NL The Netherlands

## Abstract

To date, there is no published detailed checklist with parameters referencing the DICOM tag information with respect to the quality control (QC) of PET/CT scans. The aims of these guidelines are to provide the know-how for effectively controlling the quality of PET/CT scans in multicenter studies, to standardize the QC, to give sponsors and regulatory agencies a basis for justification of the data quality when using standardized uptake values as an imaging biomarker, to document the compliance with the imaging guidelines, to verify the per protocol population versus intent to treat population, and to safeguard the validity of multicenter study conclusions employing standardized uptake value (SUV) as an imaging biomarker which is paramount to the scientific community. Following the proposed guidelines will ensure standardized prospective imaging QC of scans applicable to most studies where SUVs are used as an imaging biomarker. The multitude of factors affecting SUV measurements when not controlled inflicts noise on the data. Decisions on patient management with substantial noise would be devastating to patients, ultimately undermine treatment outcome, and invalidate the utility of SUV as an imaging biomarker usefulness. Strict control of the data quality used for the validation of SUV as an imaging biomarker would ensure trust and reliability of the data.

## Background

The variability and reproducibility of standardized uptake value (SUV) measurements and the factors affecting SUV as an imaging biomarker (IB) have been well studied and documented [1–6]. Major work has been done in the area of proposing criteria and methodology for the SUV measurements in multicenter studies [7–14]. However, limited information is available on the methodology and strategy used to verify the basics behind the factors affecting SUVs, and to date, no guidelines have been proposed offering detailed step-by-step quality control (QC) for positron emission tomography (PET) scans, with the goal to standardize reporting of imaging guidelines (IG) and protocol compliance. The aims of these guidelines are to systematize the reporting of QC in multicenter PET studies, in order to properly document it in all clinical trials involving SUVs as an IB used for the study end points, to provide investigators with the know-how for handling IG deviation related queries, and to provide the sponsors with a method to determine the size of the per protocol population (PPP) cohort. This will ensure standardized prospective imaging QC and review of scans which could be applied in most studies where SUVs are used as an IB. These guidelines are indicative in nature and may be subject to some variations depending on the complexity of the imaging indication, pharmacokinetics, and pharmacodynamics of the studied compound, the treatment technique, the number of patients enrolled in each trial, and the sponsor’s commitment.

## Background

### Key players and components

The majority of industry-sponsored clinical study implementation starts with an investigator meeting, which usually includes the patient-recruiting physician and the study coordinator at the investigating center. The aim of the imaging manual is to provide a detailed educational session for those two key players on the study protocol and the required documentation. While usually multiple departments are involved in the patient management in the clinical study (i.e., pathology, radiology, and laboratory), it is expected that the recruiting physician and the study coordinator would pass on to the rest of the departments at his/her center all relevant information, a copy of the protocol, and any specific guidelines, instructions, forms, and techniques to be used in the patient management or patient data management, including biological specimens, images, and so on. The recruiting physician is responsible to ensure that all involved parties have training on the protocol and are qualified to care for the patients in the study and manage the data.

With the increasing number of clinical studies designed to use surrogate end points such as progression free survival (PFS), more and more studies use imaging biomarkers. Imaging biomarkers can basically be divided into morphological and functional qualitative and quantitative readouts. The PET-based SUVs are semi-quantitative measurements based on functional imaging often used with a different purpose in a clinical study: patient inclusion/exclusion, response monitoring, follow-up, and as prognostic and predictive IB. In light of the increased use of well established (e.g., fluorodeoxyglucose (FDG)) and more recent PET radiopharmaceuticals (e.g., fluorothymidine) included in clinical trials, it should be clearly outlined to the PET imaging department that scan parameters will be checked during the QC of the scans by the sponsor or its designee. These details are usually not discussed in the study protocol and are not clear to the sites. Yet, often, it is the study coordinator who is tasked to reply to imaging-related queries. PET-related queries could be very technical with respect to artifacts, scan acquisition, and reconstruction parameters or could be patient- and visit-oriented. For the latter, the study coordinator should be able to provide the information without the assistance of the PET department. However, in cases where the queries quote specific scan technical parameters, usually the PET department would be the most appropriate to reply. This implies that the PET personnel at the site should be well acquainted with the imaging guidelines and the protocol end points, in order to minimize the number of queries for deviations. Furthermore, the PET personnel should be aware of the factors affecting SUVs and the implication of not following the imaging guidelines on the end points of the study. Not following the imaging guidelines could lead to a study failure [6].

**Fig. 1:**
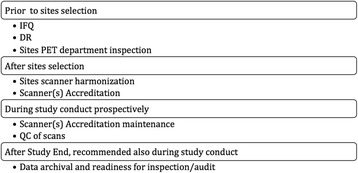
Steps ensuring data integrity

Figure 1 outlines the proposed steps to ensure data integrity for studies where SUVs are used as an IB in the clinical trial end point(s).

### Prior to the selection of sites

Imaging Facility Questionnaire **(**IFQ**)** is a questionnaire documenting the infrastructure, personnel, and technical facilities available at the site’s PET imaging department. It allows for a verification that a site meets the minimum requirements imposed by the sponsor and that it is equipped for delivering the required quality of the scans. The IFQ can be used for selecting sites for participation in a study involving specific imaging techniques and modalities.

Dummy run or dry run **(**DR**)** are scans sometimes required to be performed either on a subject or a phantom prior to enrolling patients to participate in a clinical study. If a subject scan is submitted for this purpose, a proper consent should be in place. DR is a “clinical mock test” undertaken at each site before the site is authorized to enroll patients, or at a very early phase of the study, depending on the sponsor’s decision and the study design. This mock test scan is reviewed by the sponsor or its designee, who would verify scanner performance, image quality, and consistency of acquisition/reconstruction parameters with the imaging guidelines and study protocol. The DR is usually not analyzed further. The DR may be specific to a study allowing up-front checking of the site’s ability to comply with the protocol and the IG. The DR could also identify the presence of possible ambiguities in the protocol and the IG and assist in the implementation of corrective measures to guarantee a satisfactory selection, treatment, and follow-up in study patients. DR submission also checks to ensure that scan upload is possible via electronic transfer and firewall blockage could be lifted for uploads prior to patients’ data submission.

Qualification of PET departments may vary from very detailed with an actual audit (mandatory for PET studies using on-site production of novel radiopharmaceuticals) to only providing the sites with the imaging guidelines and asking them to ascertain whether they are able to comply with the parameters. The IG contain not only detailed acquisition and reconstruction parameters for the research scans of patients in the clinical study, but also guidelines on phantom studies to obtain the necessary approval from the sponsor to use a specific scanner for the study.

### After selection of sites

PET scanner harmonization across different manufacturers has been well studied and documented [15–18]. It is required in order to homogenize scanner performance across all PET makes and models within a multicenter clinical study [19–21]. In addition to uniformity checks, SUV coefficient recovery should be checked either on a regular basis or ad hoc depending on the study needs. Several international organizations have established procedures for this in the multicenter setting across various imaging platforms [22–24]. For well-established IBs such as ^18^F-FDG, accrediting organizations have proposed a yearly check. However, if the study demands rather novel approaches, it may be reasonable to request that sites acquire a phantom scan the day of or prior to imaging a research patient, ensuring that the recovery coefficient is within the acceptable window and that corrections have to be undertaken prior to scanning a research patient, whenever needed.

Scanner accreditation must be obtained prior to the imaging of the first patient in a specific study. In a multicenter PET study, due to variability of makes and models of scanners, it is highly recommended that scanner performance is verified and documented. This is accomplished by ensuring that all PET scanners are accredited by an independent external organization (i.e., European Association of Nuclear Medicine Research Ltd., EARL; Society of Nuclear Medicine and Molecular Imaging Clinical Trial Network, SNMMI CTN; and American College of Radiology Imaging Network, ACRIN) and maintain the accreditation for the period they are participating in the study. Alternatively, regular acquisition of PET phantoms can be implemented. The phantom scans should be processed in an identical manner, using the same methodology and software. After submission to the sponsor for analysis, feedback can be given to the site with specific parameters to be used in the study. The PET scanner accreditation programs [3, 11–13] to date have known differences in assessment, e.g., recovery coefficient analysis for all scanners versus only for some which depend on the study, different software used, and difference in frequency in phantom review. Thus, it may not be appropriate for every study to use a mix of those accrediting agency certifications. For harmonization in scanner performance, it is imperative that the same methodology is used across all sites’ scanners utilized in a specific multicenter study.

### During the clinical study

It is highly recommended that a specialized PET imaging monitor visits the PET/CT department to ascertain that the PET/CT scanner is maintained as recommended by the manufacturer, all daily and periodic image quality control procedures are performed and documented. Scanner performance check should be done not just on a regular basis during the study, but also after every major software update resulting in a scanner software version change and after hardware updates/changes. During QC it should be checked that the study-qualified scanner is used for the subject’s scans in a particular study. Accreditation must be maintained for the duration of the study, or until the last patient’s scan is completed, whichever comes first. It is the sponsor’s obligation to check and ensure the site maintains its accreditation, but it is the site’s responsibility to submit the necessary data to the accrediting body and rectify any issues with image quality for the accreditation compliance. It is the site’s responsibility to promptly notify the sponsor if the accreditation can no longer be maintained, while there are still study patients scheduled for imaging on the particular scanner.

### PET scans QC

All trials using SUVs as the end points or criteria referencing SUVs, especially referring to longitudinal change such as the recent IWG 2014 lymphoma criteria [14], should implement at the minimum IG and compliance check. While the IG are part of the clinical trial protocol, often are provided segregated from the protocol. The IG detail the role of the PET department, instructions on site’s qualification to participate in the study, patient preparation, acquisition, and reconstruction parameters, when the scans have to be acquired and how to submit them.

Prospective detailed QC is recommended for all scans received, and in addition to a check of the DICOM tags for acquisition and reconstruction parameters, study pre-defined normal organ SUV_mean_ should be measured (i.e., liver, blood pool, muscle, lungs, and brain). For studies requiring the use of an accredited scanner, check should be done that such a scanner was used. If specific accredited acquisition and reconstruction parameters are required (all follow-up scans should be acquired and reconstructed identically), all scans should be checked against the required parameters. It is important that this is done almost immediately after acquisition and submission. In case of a missing reconstruction, the site would have the possibility to use the raw data still on the scanner and reconstruct and submit as required. Delay in providing QC feedback or site unresponsiveness to reconstruct, could result in loss of data, ultimately affecting the PPP. Raw data is usually overwritten in a matter of days, and it may not be possible for the site to provide further reconstructions.

#### PET series

The PET attenuation-corrected (AC), non-attenuation-corrected (NAC), and attenuation correction CT (AC-CT) series should be properly anonymized and submitted to the sponsor and inspected in detail. When reviewing the scan, it should be ascertained that it belongs to the right patient, the specified anatomy in the IG has been scanned, and the images should be assessed for possible artifacts from either the patient or the equipment (i.e., motion, dose infiltration, metal, out of field of view artifacts, truncation artifacts, misregistration, and incorrect attenuation correction). It is very important that this QC is performed by a qualified PET imaging technologist who can recognize the different artifacts and contact the sites suggesting possible remedy prior to a subsequent scan acquisition. The published guidance for industry from the Food and Drug Administration (FDA) specifies that the FDA “anticipate periodic on-site inspection by the trial’s imaging-specific monitors to assess the imaging technical compliance of each clinical site or a subset of all the sites” [25]. However, it is not clear if this is done systematically or if and how the FDA checks to ensure this recommendation is implemented. At the minimum the PETAC, NAC, and AC-CT should be submitted for every scan. However, if the sites do not submit the NAC series, the impact on the study data would be low, since this series is only used to identify questionable activity concentration during the reviews and to check for patient movement-related artifacts.

#### Compliance with the visit window schedule

The scan timing should be confirmed to ensure it is within the specified acceptable window as per the protocol. Any scans outside of pre-specified timing should clearly be identified for the sponsor to ensure proper PPP analysis.

A simple check via a region of interest (ROI) or volume of interest (VOI) with the PET software used for QC should be done to ensure that SUV measurements could be performed and that the data received contains all DICOM tag intact which are necessary and used for SUVs. Validated PET software would produce meaningful values for SUV_max_, SUV_mean_, SUV_peak_, SUV_min_, area, and so on. If some of the DICOM tag information are missing, as in a case where the site did not submit the originally reconstructed data but instead submitted post-processed data, the site should be asked to submit the original reconstructions, exported directly from the scanner. It is recommended that sites save this data as it is considered source data and is required to be saved and available upon an audit either from the sponsor or the regulatory authorities. The FDA requires the source data to be saved for at least 2 years after the last drug market authorization; however, local regulations may impose extended time, and the sites should comply with whichever is longer. Some vendor scanners produce DICOM proprietary tags with information required for the SUV measurements (e.g., Private Creator-Philips PET Private Group (7053,0010); Unknown Element-0.002348 (7053,1000)). These tags are often modified or removed by some post-processing software, DICOM-not-compliant software, or during anonymization of the patient data in DICOM. Care should be taken that the original data is saved and available, and only originally reconstructed data is used for SUV measurements.

Often the IG specify clinical data such as fasting time, medication withhold, administration or delay of scan start after a certain treatment is given, blood glucose, and other laboratory tests. This information should be checked during QC against the clinical dossier to ensure verifiability.

The following sections quote specific DICOM tags, and the assumption in this publication is that the readers have access to a DICOM tool and have a basic understanding of the DICOM standards. Table 1 includes detailed DICOM tags with explanations. We suggest using those for a longitudinal check and against the specified parameters in the IG.

**Table 1:** Detailed DICOM tags with explanations

Clinical data/DICOM field name	DICOM tag/units	QC check
Scanner make/manufacturer	(0008,0070)	Check this information against the accredited site scanner and compare to the baseline and prior scans.
Patient height/patient size	(0010,1020) (cm)	This tag has to be verified against the patient’s clinical dossier and across the longitudinal scans record. It is imperative for SUV measurements corrected for body surface area (SUV_BSA_) and lean body mass (SUV_LBM_) or glucose corrected (SUVg).
Patients weight	(0010,1030) (kg)	This tag has to be verified against the patient’s clinical dossier. It is common that, during the course of a therapy, subjects change weight. It is important that the IG mandate the use of a calibrated scale at the PET department and that each patient is weighed prior to each PET scan, and this information is correctly recorded in the PET scanner, as well as the clinical dossier. This parameter has a direct impact on the SUV, and a recent publication by Lasnon et al. [31] confirms imperative and obligatory monitoring and verification of the source data.
Scanner serial number/device serial number	(0018,1000)	Check this information against the accredited site scanner and compare to the baseline and prior scans.
Software versions	(0018,1020)	Check this information against the accredited site scanner and compare to the baseline and prior scans.
Scanner model/ manufacturer model name	(0008,1090)	Check this information against the accredited site scanner and compare to the baseline and prior scans.
Reconstruction diameter	(0018,1100) (mm)	Check this parameter to ensure that the reconstruction diameters of the PETAC and AC-CT series are equal. Smaller AC-CT reconstruction diameter could inflict truncation artifacts.
Time per bed/actual frame duration	(0018,1242) (ms)	This DICOM tag should be checked to ensure it is in compliance with the IG. Furthermore, for all subsequent scans within the same subject, the time per bed should be consistent with the baseline acquisition. This parameter is in direct relationship with the radiopharmaceutical dose a patient receives and the scanning protocol established by the vendor, but often in day-to-day clinical practice it might be acceptable to modify it; however, for patients taking part in a clinical study where SUV measurements will be performed, this parameter should remain constant.
The radiopharmaceutical injection time/radiopharmaceutical start time	(0018,1072) (h:min:s)	This information should be routinely recorded in the injection record dossier for the patient at the PET department, and this record is considered source data. The injection time is not the same as the assay time. Those two always differ, unless an automatic injection is used. The injection time is usually entered manually into the acquisition protocol of the scanner. Those two records should match explicitly and should refer to the actual injection time and not the dose assay time. This requires synchronization of all clocks at the PET department—the hot lab, the injection room, the control room, and the scanner itself. Any mismatch between the clocks should be rectified immediately and, if not possible, prior to scanning patients, it should be documented and reported to the sponsor. Based on the experience of the co-authors of these guidelines, up to 1 min difference between all clocks is acceptable and it would not have a major impact on SUV measurements for isotopes such as ^18^F. For some scanner makes and models, the dose is decay corrected automatically and the information is captured in the proprietary DICOM tags.
Collimator type	(0018,1181)	Ensure same collimator applied, which would affect image quality and quantification.
Acquisition mode (2D/3D)	(0018:9732)	This tag may or may not be present to check, but it is imperative that the mode of acquisition is consistent between baseline and all follow-up scans. While in low BMI patients, the mode of acquisition may not significantly affect the visual readout; it could affect the SUV. For patients with high/obese BMI, both visual and SUV readouts could be affected more severely.
Uptake time/N/A	N/A	UT is another parameter very well documented affecting SUVs. The UT should be checked in the DICOM tags by calculating the difference between the Radiopharmaceutical Injection Time, and the earliest scan start time [earliest acquisition time (0008.0032)-radiopharmaceutical start time (0018,1072)]. Furthermore, it should be checked for compliance with the IG for the baseline and all subsequent scans. Some post-processing software use the Series Start time tag. The authors of these guidelines suggest not to use the Series Start time as this tag could be erroneously changed during export of the data from a PACS or another stand-alone post-processing software.
Radiopharmaceutical dose/radionuclide total dose	(0018.1074) (h:min:s)	The net injected radiopharmaceutical dose recorded in the patient’s clinical dossier/PET department records should match the dose in the DICOM tag. It is usually entered manually into the scanner, and it should be reliable as a source data. It is acceptable within the nuclear medicine practice to use ± 10% variability from the prescribed dose and to base the dose on the patient weight. Thus, for all follow-up scans, in the absence of drastic weight change, the net injected dose should be within ± 10% of the baseline net injected dose. Greater variability could affect the available radiopharmaceutical in the body and influence the SUV comparability artificially. In the case a patient incurs weight change, which results in greater than 10% dose change compared to the baseline scan, this should be recorded and available during QC for verification. Dose assay time is not the same as dose injection time, and since those two always differ, the net injected dose should be decay corrected accordingly to account for the difference between the time of assay and the actual time of injection. To calculate manually the net injected dose, the residual activity in the syringe with any tubing (dose “N” at time “n”) is subtracted from the assayed dose (dose “A” at time “a”) and decayed accordingly to account for the difference in time between “a” and “n”. This is vital for radioisotopes with very short half-life.
Scanning position	N/A	Patient position should remain constant across all scans unless the position is changed due to the inability of the patient to comply. Often, during the course of treatment, subjects may experience significant weakness and inability to keep their arms up or lay supine. If patient’s position is changed at a follow-up scan compared to the baseline, there should be a reasonable explanation about this in the clinical dossier and provided to the sponsor.
Scan direction	N/A	The acquisition time (0008.0032) of the most superior and most inferior transaxial slice is used to determine the scan direction. The scan direction (caudal vs. cranial) should remain consistent with the baseline scan. While a single-bed-position or two-bed-position acquisition scan direction change would not influence greatly the SUV measurements, multiple-bed-position acquisition especially for high-grade tumors could show large changes in SUV measurements due to change in scan direction. By itself, this factor may not be significant enough to influence a patient’s management and change the patient’s classification from one category to another, but in combination with other factors, the SUVs could be influenced enough to bring artificial noise.
Reconstruction algorithm/reconstruction method	(0054,1103)	Usually, during the scanner qualification process, the acquisition and reconstruction parameters are established for each scanner to be used in a specific clinical study. In the frame of a specific clinical trial, when accreditation of scanners is mandated, it is specified what manufacturer availed parameters should be used for each patient taking part of the study. Those parameters may be different from one to another study. It is best that the longitudinal PET scans for all subjects in a given study are acquired and reconstructed with the same exact parameters and be cross-checked for all scans, including acquisition and reconstruction field of view (FOV), mode of acquisition (e.g., 2D vs. 3D), matrix size, slice thickness, reconstruction algorithm, and used corrections.
Corrections applied/corrected image	(0028,0051)	
Matrix size/rows & columns	(0028,0010) (0028,0011)	
Slice thickness	(0018,0050)	

*N/A* not applicable

Recent treatment interventions or health conditions may affect SUV, e.g., a significant difference in SUV_max_-induced by recent chemotherapy [26–28]. When applicable, this information should be available and taken into consideration during QC and in the interpretation of longitudinal PET scans.

Some of the factors noted above, taken by themselves, may not significantly influence SUV measurements, yet a combination of them could result in an erroneous patient category assignment, e.g., eligibility, stratification, and crossover in study arms. Some factors, however, such as the use of point spread function (PSF) within the iterative reconstruction algorithm, could double the change in SUV_max_ measurements in pathology [29], while SUV_mean_ in normal organ such as the liver will remain almost constant.

It is important to note that not all systems or vendors provide sufficient information in the DICOM tags about reconstruction type, settings, and/or image processing. Thus, images that have been reconstructed and/or processed differently cannot always be distinguished based on the metadata alone. This only underlines the importance of ensuring that reliable and high-quality data is analyzed.

**Fig. 2:**
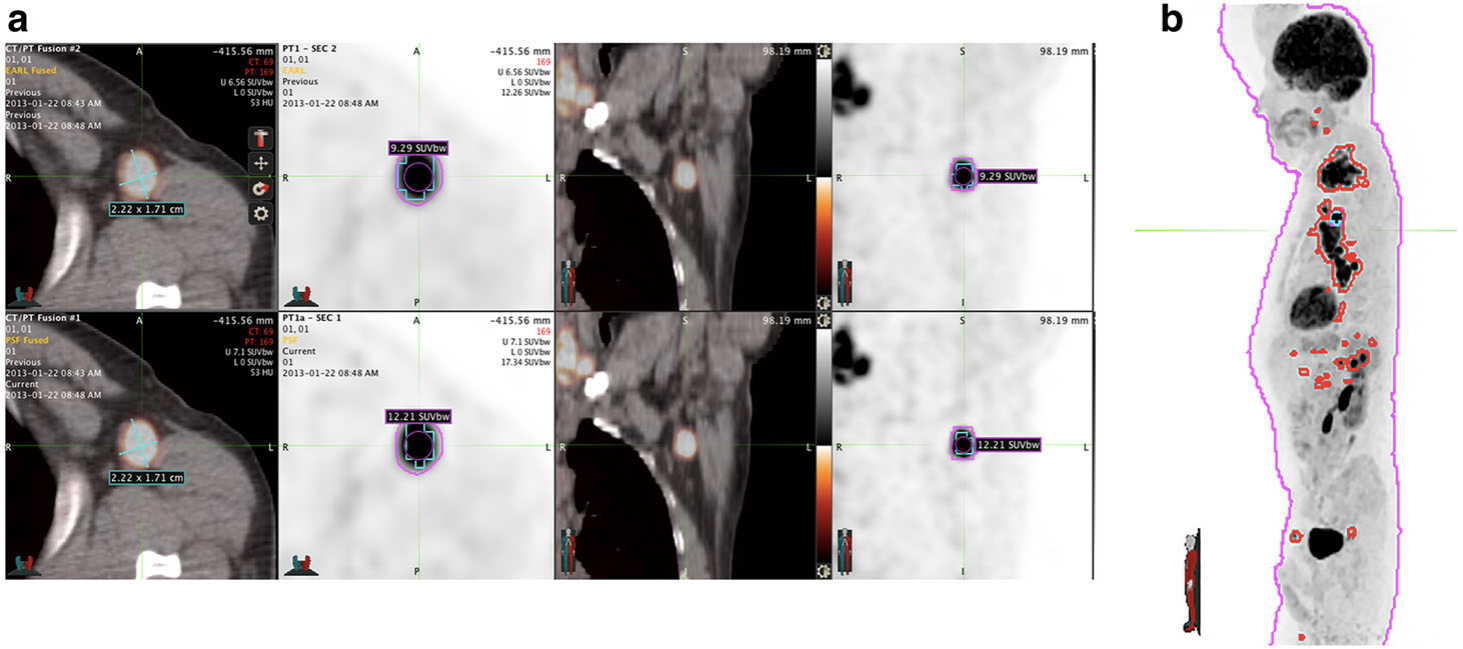
**a** Single lesion volume of interests (1) for entire lesion with (2) RECIST measurement and (3) 40% SUV threshold. **b** Whole body tumor burden maximum intensity projection-segmented based on the AC-CT, contour was then copied to the two PETAC reconstructions and sub-segmented to include only SUV values above 2.5 and normal tissue uptake was subtracted (i.e., brain and kidney)

The use of different reconstruction parameters and the effect on SUVs have been discussed extensively already. Magnitude of effect is variable, and it depends on the different make and model scanners used, as well as the various reconstruction parameters applied. Illustration of such effects is shown here based on a patient scan as follows:

- Table 2 shows the values of SUVs (corrected for body weight (bw), lean body mass (lbm), and body surface area (bsa)) of a single lesion, large enough to be selected as a target lesion, and a whole body total glycolytic activity of total body tumor burden for two different reconstructions
- Table 3 shows the values of SUV (corrected for bw, lbm, and bsa) of normal tissue (blood pool, liver, muscle, lung) using the site standard of care PSF reconstruction and EARL reconstruction
- Figure 2a (single lesion) and b (whole body tumor burden maximum intensity projection) shows the images. In Fig. 2b, the whole body was first segmented based on the AC-CT to include skin to skin anatomy in 3D. The VOI from this section is then copied to the two PETAC reconstructions and sub-segmented to include only SUV values above 2.5, and normal tissue uptake was subtracted (i.e., brain and kidney activity).

**Table 2:** Values of SUVs (corrected for bw, lbm, and bsa) of a single lesion, large enough to be selected as target lesion per RECIST, and a whole body total glycolytic activity for tumor burden for two different reconstructions (site standard of care PSF and EARL)

	body weight-corrected SUV						lbm-corrected SUV						bsa-corrected SUV					
Statistic	ROI-1	ROI-1	ROI-1 bw (above 40%)	ROI-1 bw (above 40%)	Whole body (above 2.5 SUV_bw_)	Whole body (above 2.5 SUV_bw_)	ROI-1	ROI-1	ROI-1 (above 40% SUV_lbm_)	ROI-1 (above 40.% SUV_lbm_)	Whole body (above 2.5 SUV_lbm_)	Whole body (above 2.5 SUV_lbm_)	ROI-1	ROI-1	ROI-1 (above 40% SUV_bsa_)	ROI-1 (above 40.% SUV_bsa_)	Whole body (above 2.5 SUV_bsa)_	Whole body (above 2.5 SUV_bsa_)
Reconstruction	PSF	EARL	PSF	EARL	PSF	EARL	PSF	EARL	PSF	EARL	PSF	EARL	PSF	EARL	PSF	EARL	PSF	EARL
Max (SUV) value	17.34	12.26	17.34	12.26	18.04	13.07	14.08	9.95	14.08	9.95	14.64	10.61	0.44	0.31	0.44	0.31	0.46	0.33
% change	41.47	–	–	–	38.04	–	41.47	–	–	–	38.04	–	41.47	–	–	–	38.04	–
Mean (SUV) value	5.94	5.16	11.11	7.65	5.24	4.78	4.82	4.19	9.02	6.21	4.26	3.88	0.15	0.13	0.28	0.19	0.13	0.12
% change	15.14	–	–	–	9.58	–	15.14	–	–	–	9.58	–	15.14	–	–	–	9.58	–
PERCIST SUV peak	12.21	9.29	–	–	–	–	9.91	7.54	–	–	–	–	0.31	0.24	–	–	–	–
% change	31.37	–	–	–	–	–	31.37	–	–	–	–	–	31.37	–	–	–	–	–
Total glycolytic activity (SUV*ml) value	44.83	38.93	25.61	25.44	1329.13	1212.89	36.38	31.6	20.78	20.65	1078.75	984.41	1.14	0.99	0.65	0.65	33.77	30.82
% change	15.14	–	–	–	9.58	–	15.14	–	–	–	9.58	–	15.14	–	–	–	9.58	–
Volume (ml) value	7.54	7.54	2.3	3.33	253.49	253.49	7.54	7.54	2.3	3.33	253.49	253.49	7.54	7.54	2.3	3.33	253.49	253.49

**Table 3:** Values of SUVs (corrected for bw, lbm, and bsa) of normal tissue (blood pool, liver, muscle, lung) of two different reconstruction (site standard of care PSF and EARL)

	Body weight-corrected SUV								lbm-corrected SUV								bsa-corrected SUV							
Normal tissue	Aortic arch	Aortic arch	Liver	Liver	Muscle	Muscle	Rt lung	Rt lung	Aortic arch	Aortic arch	Liver	Liver	Muscle	Muscle	Rt lung	Rt lung	Aortic arch	Aortic arch	Liver	Liver	Muscle	Muscle	Rt lung	Rt lung
Reconstruction	EARL	PSF	EARL	PSF	EARL	PSF	EARL	PSF	EARL	PSF	EARL	PSF	EARL	PSF	EARL	PSF	EARL	PSF	EARL	PSF	EARL	PSF	EARL	PSF
Max (SUV) value	1.71	1.85	2.34	2.59	1.04	1.13	0.6	0.66	1.39	1.5	1.9	2.1	0.84	0.92	0.49	0.53	0.04	0.05	0.06	0.07	0.03	0.03	0.02	0.02
% change	–	7.72	–	10.76	–	8.56	–	9.42	–	7.72	–	10.76	–	8.56	–	9.42	–	7.72	–	10.76	–	8.56	–	9.42
Mean (SUV) value	1.41	1.39	1.73	1.73	0.87	0.89	0.41	0.4	1.14	1.13	1.41	1.4	0.71	0.72	0.33	0.33	0.04	0.04	0.04	0.04	0.02	0.02	0.01	0.01
% change	–	−1.02	–	−0.34	–	2.22	–	−2.42	–	−1.02	–	−0.34	–	2.22	–	−2.42	–	−1.02	–	−0.34	–	2.22	–	−2.42
Median (SUV) value	1.38	1.34	1.73	1.71	0.87	0.89	0.4	0.39	1.12	1.09	1.4	1.39	0.71	0.72	0.33	0.32	0.04	0.03	0.04	0.04	0.02	0.02	0.01	0.01
% change	–	−2.52	–	−0.63	–	1.75	–	−3.23	–	−2.52	–	−0.63	–	1.75	–	−3.23	–	−2.52	–	−0.63	–	1.75	–	−3.23
Volume (ml) value	4.27	4.27	13.79	13.79	4.24	4.24	4.28	4.28	4.27	4.27	13.79	13.79	4.24	4.24	4.28	4.28	4.27	4.27	13.79	13.79	4.24	4.24	4.28	4.28

Informed consent was obtained from all individual participants whose data was used in this article.

SUV measurements were performed using MIM Vista software (MIM Software Inc., Cleveland, OH USA). In this case, the data presented in the DICOM tags is identical as to acquisition and reconstruction parameters. However, as shown via the SUV measurements, it is clear that the reconstructions are different.

Radiopharmaceutical administration should be performed only via saline-tested and well patent IV access. Extravasation is rare, however, in the case of observed extravasation affecting the study quality, and ultimately rendering the quantification incorrect, scan repeat might be necessary when possible.

Changes in SUV used for patient management, or for clinical studies end points, should be based on true, high-quality data, verified to ensure that technical factors have been excluded as a source of error. Without detailed QC of the parameters described in these guidelines, it is possible that treatment allocation is made merely based on factors, which could mislead one way or the other, leading either in change in treatment strategy or not. In both instances, the results are devastating to patients. As a whole, increased noise in the data of a clinical study and questionable quality of the applied methods, undermine the validity of study conclusions and may lead to a clinical study failure or to an ultimate positive result, which contains invalid source data. Rarely, if ever, the regulatory authorities check to ensure that for surrogate end points the data presented is, in fact, free of technical artifacts. While the latest FDA guidelines from March 2015 [30] include more stringent points on quality, it is not known or clear if any of the additional suggestions made will be implemented, checked, and complied with in the future.

### After study completion

After study completion, it must be ensured that the data are properly archived and ready for inspection by either the regulatory agencies or the sponsor. Any changes to the digital data (i.e., DICOM tag update) must be done in an audit trail fashion, ensuring the ability to track the changes per the specified regulations.

Implementing the steps suggested in Fig. 1 as detailed above, prior and after site selection, during and at end of a study, would ensure the integrity of the data, the scientific value, and boost trust in results and would ultimately help patients in the fight of disease. Verifiable quality data, even in a small number of patients, is the future of IB use as surrogate end points in clinical trials.

## Summary

### Concluding remarks

While daily clinical standard of care variations is a fact, bringing noise in the day to day practice, validation of SUVs as IB aims to set out an international standard to be relied on; thus, the quality of the data for the IB must be held to the highest standards. Assuring the quality of imaging data as proposed in these guidelines aim to safeguard the validity of imaging data and study conclusions, which is relevant for scientific journals, the regulatory authorities, and the sponsors. Implementation of rigorous QC procedures ensures that basic data affecting SUVs are verified prior to readouts. In the absence of QC, readouts could be merely a reflection of technical factors, and conclusions about the validity of PET as an IB may be inappropriate.
